# Chromosome Conformation Capture Uncovers Potential Genome-Wide Interactions between Human Conserved Non-Coding Sequences

**DOI:** 10.1371/journal.pone.0017634

**Published:** 2011-03-07

**Authors:** Daniel Robyr, Marc Friedli, Corinne Gehrig, Mélanie Arcangeli, Marilyn Marin, Michel Guipponi, Laurent Farinelli, Isabelle Barde, Sonia Verp, Didier Trono, Stylianos E. Antonarakis

**Affiliations:** 1 Department of Genetic Medicine and Development, University of Geneva Medical School and University Hospitals of Geneva, Geneva, Switzerland; 2 FASTERIS SA, Plan-les-Ouates, Switzerland; 3 Global Health Institute, School of Life Sciences, Ecole Polytechnique Fédérale de Lausanne (EPFL), Lausanne, Switzerland; Bellvitge Biomedical Research Institute (IDIBELL), Spain

## Abstract

Comparative analyses of various mammalian genomes have identified numerous conserved non-coding (CNC) DNA elements that display striking conservation among species, suggesting that they have maintained specific functions throughout evolution. CNC function remains poorly understood, although recent studies have identified a role in gene regulation. We hypothesized that the identification of genomic loci that interact physically with CNCs would provide information on their functions. We have used circular chromosome conformation capture (4C) to characterize interactions of 10 CNCs from human chromosome 21 in K562 cells. The data provide evidence that CNCs are capable of interacting with loci that are enriched for CNCs. The number of trans interactions varies among CNCs; some show interactions with many loci, while others interact with few. Some of the tested CNCs are capable of driving the expression of a reporter gene in the mouse embryo, and associate with the oligodendrocyte genes OLIG1 and OLIG2. Our results underscore the power of chromosome conformation capture for the identification of targets of functional DNA elements and raise the possibility that CNCs exert their functions by physical association with defined genomic regions enriched in CNCs. These CNC-CNC interactions may in part explain their stringent conservation as a group of regulatory sequences.

## Introduction

The sequencing and current annotation of the human genome revealed that it contains about 21500 protein coding genes (Ensembl build GRCh37) [1], [2]. However, the overwhelming majority of the human genome is composed of non-coding DNA whose function has not been thoroughly investigated. Interestingly, approximately 5% of the human genome is conserved in other eutherian mammals [3], [4]. The recent analysis of DNA topography conservation, rather than nucleotide sequence, suggested that up to 12% of the human genome could be under evolutionary constraint [5]. A significant number of CNCs (conserved non-coding sequences) are found in gene-poor regions of the genome, suggesting that these large intergenic regions have maintained a function throughout evolution [6], [7]. The function of most CNCs remains elusive although recent studies have begun to assign function to a fraction of them. Some CNCs appear to be transcriptional enhancers *in vivo* [8], [9], [10], [11] although their deletion does not appear to be detrimental for mouse development in one study [12], despite evidence that CNCs are maintained by negative selective pressure [13], [14]. However, the importance of CNCs in disease has been documented in several disorders including preaxial polydactyly [15], [16], [17], human NSCL/P (non syndromic cleft lip with or without cleft palate) [18] holoprosencephaly [19] and Pierre Robin sequences (a subgroup of cleft palate) [20]. Furthermore, CNCs might act as silencer elements [21]. It was also proposed by computational analysis that as much as 10% of CNCs correspond to matrix-attachment regions (MARs) [22]. Finally CNCs might be involved in other cellular processes that remain to be determined.

Most functional studies of CNCs performed to date utilized various enhancer essays in order to test their potential role in gene regulation [8], [9], [21]. Although successful, these approaches are not suited for the determination of the functions of CNCs that do not behave as transcriptional enhancers and provide little information on the genes they regulate, as enhancers can act over long distances [16]. Here, we took a different approach aimed at the analysis of physical interactions between CNCs and the rest of the genome using circular chromosome conformation capture (4C) [23], [24]. We argue that the identification of “CNC interacting regions” (CIRs) could provide valuable information on the function of the tested CNCs and on the target genomic regions they interact with. In this paper we describe the CIRs of 10 CNCs from human chromosome 21 (HSA21).

## Results and Discussion

### Proof-of-principle: the human β-globin locus control region

**Figure 1:**
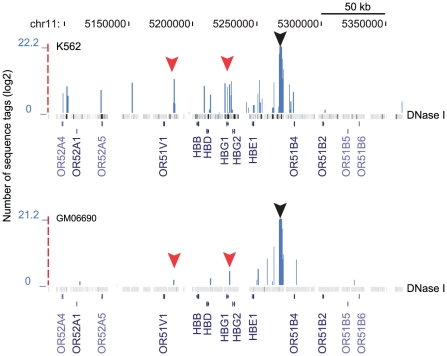
Interaction map of the beta-globin locus control region (LCR). The blue bars correspond to the number of sequence tags (log 2 transformed) found in the library generated with HS5 (black arrow) in the indicated cell line. The red arrow corresponds to regions of the locus where interactions were described in earlier studies. The genes found in the region are displayed at the bottom of the map. The image was generated with the UCSC genome browser (http://genome.ucsc.edu/). The DNaseI hypersensitivity heat map was obtained through the UCSC browser.

Chromosome conformation capture (3C) was first developed in order to map interactions between neighboring chromosome loci [25] and was later adapted to genome-wide applications (4C) [23], [24]. Since we have no prior-knowledge of the interaction maps of CNCs we carried out a proof-of-principle 4C experiment using the hypersensitive site 5 of the human β-globin locus control region (HS5-LCR), whose interactions in *cis* are partially known [26]. We have confirmed previous observations of interactions between HS5 and the promoter region of the gamma globin gene HBG1, as well as with a region near the 3′HS in K562 cells where the locus is active (Figure 1). Moreover, additional interactions were mapped to various DNase I hypersensitive sites surrounding the β-globin gene locus [4]. As expected, these interactions are lost or dramatically reduced in B-lymphoblastoid cell line (GM06990) where the locus is inactive. Interestingly, over 99.99% of the sequence tags map to the same chromosome as the β-globin locus (HSA11) reflecting the robustness and specificity of the method. These results show that this approach can be applied to the analysis of HSA21 CNCs.

### Interaction maps of 10 human chromosome 21 CNCs in K562 cells

We have sequenced 4C libraries for 10 CNCs in duplicate with non-synchronized myelogenous leukemia K562 cells. Reproducibility of the duplicates ranges from 43.8% to 88% with the exception of CNC10 (9.2%) (Table S4). Nine CNCs are located within a 700 kbp region encompassing the OLIG1 and OLIG2 genes on chromosome 21 ENCODE region ENm005 [4] (Figure S1 and Table S1). CNC10 maps upstream of PSMG1 and BRDW1 genes in ENCODE region ENr133. All CNCs but CNC1 (intronic) are intergenic. Conservation score of the CNCs ranges from 525 to 710 (PhastCons most conserved elements [3]) (Table S1). Additionally, libraries were generated with a selection of 8 non-CNCs (nonCNC2, 3, 4 and 5 map to ENCODE region ENm005) (Figure S1 and Table S1). The chromosome coordinates of all DpnII fragments as well as their abundance is available as supplementary data (File S1).

**Figure 2:**
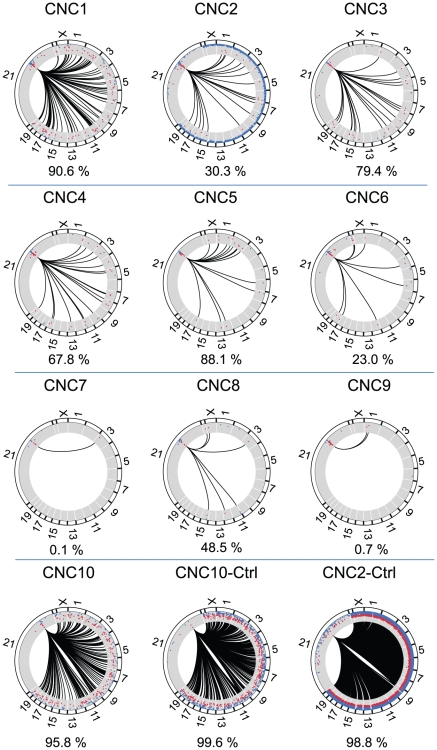
Circular representation of the interactions identified for all CNCs. All chromosomes are drawn to scale with the exception of chromosome 21. Lines are connecting the specified CNC with the DpnII fragments observed at least 50 times in *trans*. Dots in blue in the scatter plot inside the circles represent sequences observed less than 50 times, whereas red dots correspond to sequences observed at least 50 times (in *cis* or *trans*). The images were generated with the circos software package (http://mkweb.bcgsc.ca/circos/) [42]. The numbers shown below each interaction map correspond to the percentage of trans CIRs (threshold of 50 observations).

**Figure 3:**
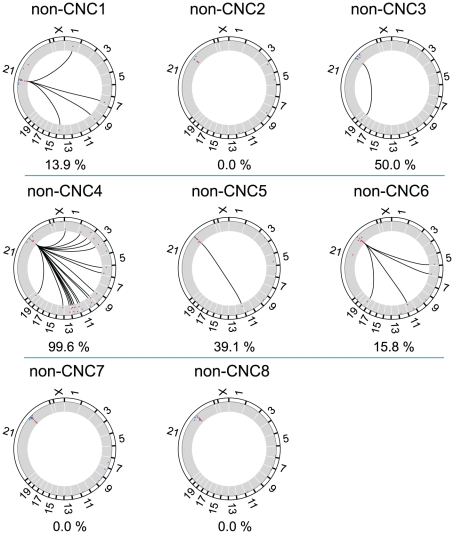
Circular representation of the interactions identified for all nonCNCs. All chromosomes are drawn to scale with the exception of chromosome 21. Lines are connecting the specified CNC with the DpnII fragments observed at least 50 times in *trans*. Dots in blue in the scatter plot inside the circles represent sequences observed less than 50 times, whereas red dots correspond to sequences observed at least 50 times (in *cis* or *trans*). The images were generated with the circos software package (http://mkweb.bcgsc.ca/circos/) [42]. The numbers shown below each interaction map correspond to the percentage of trans CIRs (threshold of 50 observations).

We have observed both *cis* (CIRs on the same chromosome as the CNC) and *trans* CIRs for all CNCs analyzed (Figure 2). However, and unlike the interactions of the β-globin LCR (see above), a substantial number of CIRs are found in *trans*. Individual CNCs display various ratios of *trans*-CIRs ranging from 0.1% (CNC7) to 95.8% (CNC10) of the total number of mappable sequence tags (Figure 2). The DpnII fragments flanking directly the CNCs were not considered as these are in excess in the library, due either to partial DpnII restriction or to a proximity effect [25]. As control, we have sequenced two additional libraries generated on purified genomic DNA (fully digested and ligated) in the absence of cross-linking for CNC2 and CNC10 (Figure 2). Under these conditions, the number of *trans*-CIRs increased to 98.8% and 99.6% respectively, which is near the number of non chromosome 21 DNA fragments expected in a random library (about 99% of the genome). We have sequenced 4C libraries from 8 non-CNCs in order to assess whether single copy non-conserved sequences behave differently than CNCs. Although some non-CNCs are capable of interactions in *trans*, 3 non-CNCs (non-CNC2, 7, 8) do not associate with any loci on other chromosomes (Figure 3). Overall these data show that CNCs have the capacity to make extensive interactions with loci on other chromosomes.

### CNCs likely interact with CNCs

**Figure 4:**
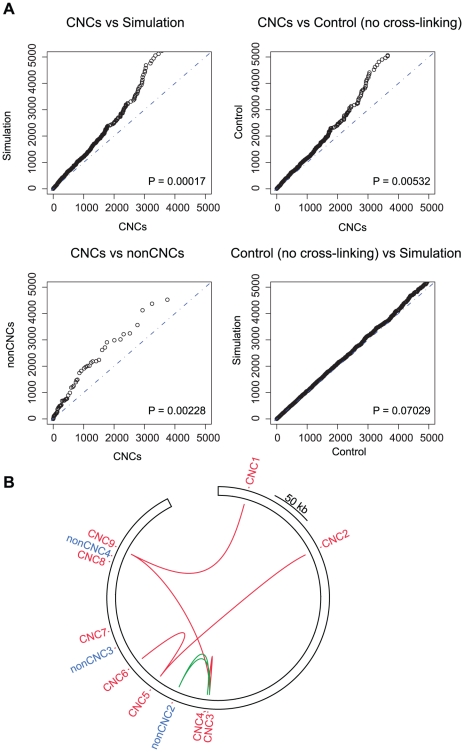
Enrichment of conserved regions. A) Comparisons of the quantile distributions of the distances (bp) between all DpnII fragments and their nearest conservation block. 10000 DpnII fragments were sampled from the genome for the “simulation” distribution. The dashed lines correspond to the expected plot if the distributions are equal. The P-value (two sample Wilcoxon test) of the divergence between two distributions is displayed at the bottom-right of each plot. B) Lines are connecting DpnII fragments that are within 5 kb of the interrogated CNC and non-CNCs in any of the other libraries analyzed in this study. The red lines connect interacting CNCs whereas the green lines represent interactions observed between CNCs and non-CNCs. The image was generated with the circos software package (http://mkweb.bcgsc.ca/circos/) [42].

Conservation of DNA elements by negative selection, such as CNCs, suggests that these sequences have maintained a function during evolution. We hypothesized that functional CIRs are also conserved and thus tested whether the CIRs are enriched for other conserved sequences. The distance (bp) between all DpnII fragments and their respective nearest non-exonic conservation block was measured center to center. The list of non-exonic conservation blocks was obtained after the subtraction of conserved exons (UCSC browser exoniphy track) to the PhastCons most conserved elements (17-way Vertebrate Multiz Alignment) [3]. This distance was measured for all DpnII fragments sequenced at least 50 times. Interestingly, there are significantly shorter distances between the DpnII fragments in the CNC libraries and the nearest conservation block in comparison with either a simulation (wilcoxon rank test P = 0.00017), or control CNC libraries in the absence of formaldehyde cross linking (P = 0.00532) (Figure 4A). The control libraries should be totally random since they should not enrich DpnII fragments in proximity to the baits. As expected there is no strong statistical difference between the simulation and the control experiments (P = 0.07029). It could be argued that the region of chromosome 21 analyzed in this study, rather than its specific CNCs, has a propensity to interact with other conserved regions. In order to rule out this possibility we have performed a similar analysis with 8 non-CNC libraries generated from the same genomic neighborhood (Table S1 and Figure S1). Indeed, CNCs interact with regions closer to CNCs when compared to non-CNCs (P = 0.0022) (Figure 4A), arguing against a region-specific effect. Overall, these results provide a first indication that CNCs can interact or co-localize with CNCs genome-wide. Moreover, the latter have globally a slightly higher score of conservation when compared both with our simulation (P = 0.012), non-CNCs (P = 0.078) and control experiments in the absence of cross-linking (P = 0.044) (Figure S2).

Since some CNCs tend to interact with regions near CNCs, we next investigated whether similar interactions exist in *cis* among the 9 CNCs and 3 non-CNCs spanning 0.7 Mb of the ENCODE region ENm005 [4]. We tested whether a DpnII fragment within 5 kb of the interrogated CNC is observed in the other 12 libraries (Figure 4B). We only considered fragments that were sequenced at least 50 times. This is indeed the case for several CNCs, suggesting that CNCs in this region either interact physically or co-localize in the nucleus. For instance, DpnII fragments within 5 kb of CNC5 are found in the 4C library generated with CNC6 and CNC2. Those interactions are not observed with other CNCs such as CNC7 and CNC8. Three non-CNCs (2, 3, and 4) are located in the same region. Although non-CNC2 library contained DpnII fragments within 5 kb of nearby CNC3 and CNC4, none of the non-CNCs make contacts with more distant CNCs, as observed between CNC9 and CNC1 (about 0.7 Mb apart). A detailed map of all cis-interactions (CNC1 to CNC9) within a 1.4 Mb window is displayed in figure S3.

These results provide initial evidence that the function of CNCs is potentially mediated by their interactions with other conserved regions either in *cis* or genome-wide. A CNC in each diploid cell could have two potential interactions at any given time and therefore the CIRs we detected represent cumulative interactions observed within a cell population. In a dynamic cell population, it is possible that only a fraction of these CIRs are functional. In addition, the stability of these interactions could change over the course of the cell cycle. It would be interesting to study the dynamics of these interactions during cell cycle.

### Potential interactions of CNCs with protein-coding genes genome-wide

The observation that CNCs are capable of numerous interactions suggests that some CNCs might participate in the co-regulation of groups of protein-coding genes in response to specific cellular signals. In order to test this hypothesis we have generated a list of protein-coding genes located within 10 kb of all DpnII fragment tags (minimum threshold of 50 sequence tags) in each CNC library. The overlaps between these genes and various data sets were computed using the Molecular Signatures Database (MSigDB) [27]. Most CNCs show CIRs located near genes enriched for specific functions (Table S3). For example the library generated with CNC5 contains fragments in the vicinity of 14 genes. Among these, POLA1, RFC3 and NHEJ1 are involved in DNA repair (P = 1.27×10^−3^). Similarly, 3 out of 10 genes within 10 kb of a DpnII fragment associated with CNC6 are implicated in vesicle-mediated transport (ITNS1, SYNJ1 and DOPEY2; P = 1.28×10^−3^). We next asked whether DpnII fragments within a given library of interacting fragments share a common DNA motif using MEME [28]. Although DNA motifs were identified none appeared to be significantly enriched. Overall these data suggest that some CNCs may participate in the co-regulation of a subset of functionally related genes.

### Putative role of CNC7 and CNC8 in the regulation of OLIG genes

**Figure 5:**
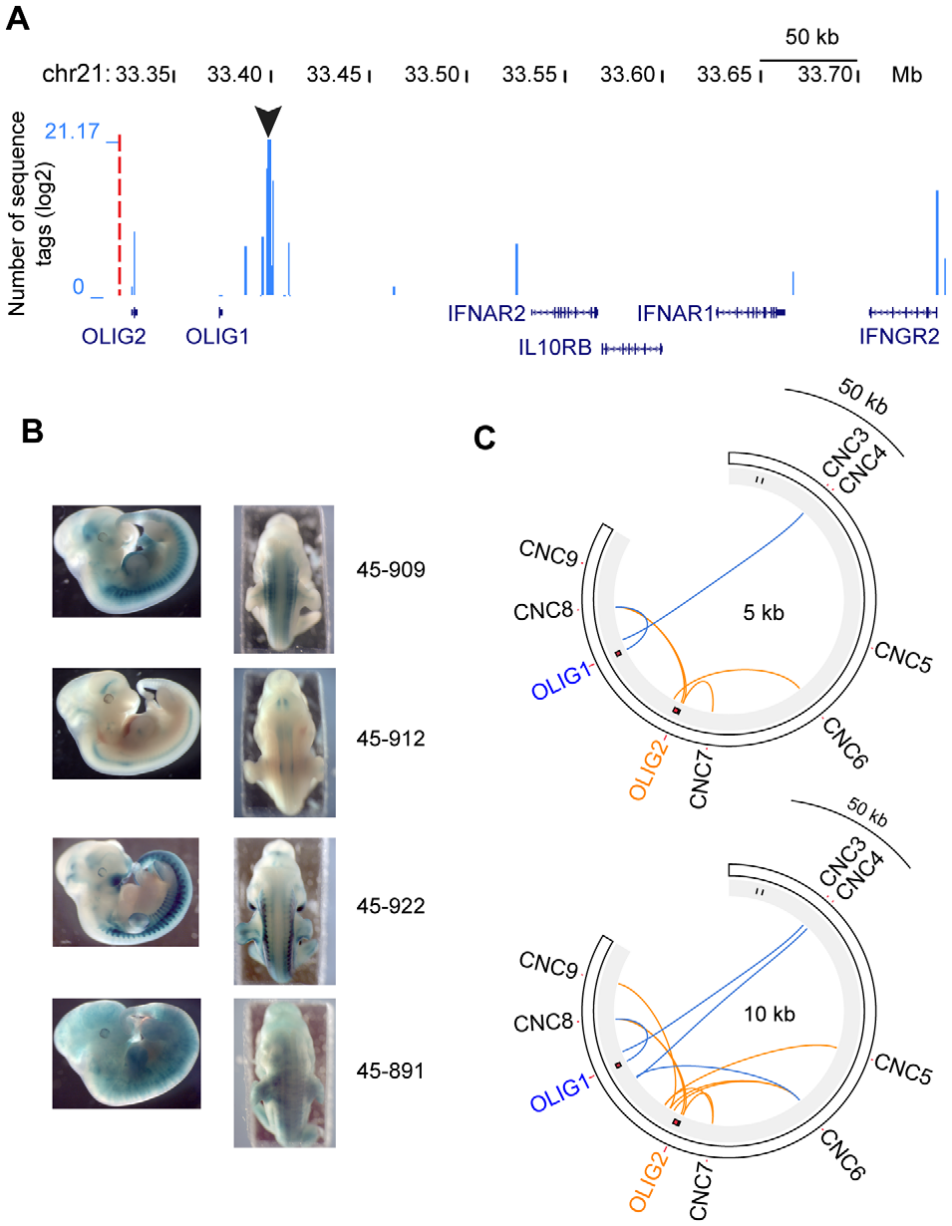
Several CNCs are potential regulators of the Olig genes. A) Interaction map of CNC8 (black arrow) with regions in *cis* in K562 cells. The blue bars correspond to the number of sequences tags (log 2 transformed) found in the library generated with CNC8. The genes in the region are displayed at the bottom of the map. The image was generated with the UCSC genome browser (http://genome.ucsc.edu/). B) Sagital and dorsal view of mouse embryos at E11.5 stained for LacZ expression after injection of CNC8 fused to LacZ in a lentiviral vector. 4 of the 45 embryos that have integrated the construct are shown here. C) Lines are connecting CNCs with fragments within their libraries that are less than 5 kb (upper) or 10 kb (lower) away from either OLIG1 (blue) or OLIG2 (orange). Images were generated with the circos software package (http://mkweb.bcgsc.ca/circos/) [42].

Careful analysis of *cis*-interactions for CNC8 showed that the latter interacts with the single coding exon of the OLIG2 gene, as well as with regions near the interferon receptor genes IFNAR2, IFNAR1 and IFNGR2 (Figure 5A). These interactions suggest that CNC8 might be involved in the regulation of some of these genes, although none of them are transcriptionally active in K562 cells. In order to investigate further the potential role of CNC8 in gene regulation, we have evaluated the ability of the orthologous mouse CNC to enhance gene expression during development. Fertilized mouse embryos were injected with a lentiviral vector with the mouse syntenic region (chr16: 91179971-91182599, assembly Mm8) containing CNC8, fused to a LacZ reporter gene. The expression pattern of the β-galactosidase reporter gene was assessed at embryonic day 11.5 (E11.5) (Figure 5B). Among 45 embryos displaying at least one integration event of the transgene, 33 (73%) were LacZ positive. Eight embryos show expression of the transgene in the somites (Figure 5B, embryos 45–909 and 45–922) indicating that the region containing the mouse orthologue of CNC8 has the ability to drive gene expression *in vivo*. The pattern of expression driven by CNC8 is not restricted to the somites since LacZ staining is also observed in the neural tube of 5 embryos (Figure 5B, embryos 45–912 and 45–922). A substantial number of embryos (8 out of 33) display ubiquitous staining (Figure 5B, embryo 45–891). Moreover, expression is observed in a few embryos in the limb bud and in the brain, albeit less frequently. The expression of LacZ in the neural tube, as well as in parts of the brain, suggest that the target of CNC8 could be OLIG2 and/or OLIG1 whose murine patterns of expression are similar to the LacZ activity observed [29].

Overall these results suggest that CNC8 is a potent enhancer of transcription although its specificity was different among embryos. The number of integrations as well as the genomic site of integration could explain the differences in the LacZ expression pattern among embryos. Alternatively, CNC8 could be a general regulator of gene activity, whose temporal and spatial specificity requires other DNA elements and a particular genomic context. If indeed CNC8 requires other DNA elements to function properly, other CNCs in the region might provide this additional layer of regulation. Moreover, as described above, CNCs in the region have the tendency to interact with each other (Figure 4). Thus, we tested whether other CNCs analyzed in this study showed interactions with or in the vicinity of OLIG2 (Figure 5C). Indeed, CNC6 and CNC7 (in addition to CNC8) display interactions within 5 kb of OLIG2. Two additional CNCs (CNC5, CNC9) have CIRs within 10 kb of that gene despite of the fact that these are almost 0.2 Mb apart. CNC7, which is located about 16.5 kb upstream of OLIG2, show an interaction with a DpnII fragment 0.2 kb downstream of its transcription start site. Most interestingly, the mouse orthologue of CNC7 enables activation of a LacZ reporter in the neural tube as well as in the posterior diencephalon at E11.5 [30].

There are two lines of evidence suggesting that CNC7 and CNC8 are potential transcriptional regulators of OLIG2. First, we observe interaction between these CNCs and OLIG2. Second, these CNCs act as enhancers in regions of the mouse embryo that co-localize with the natural expression of OLIG2 [31]. Recent experiments combining enhancer identification by ChIP-seq against p300 and mouse transgenesis have shown that a region encompassing CNC8 drives the expression of a reporter gene in the mouse embryo forebrain at E11.5 [11]. However, we were surprised to observe these interactions in K562 cells where OLIG2 is silent or expressed at very low levels, indicating that these regions are not functional enhancers in this cellular context. Chromatin looping of these regions over the promoter of OLIG2 could either be repressive or non-productive, akin to a poised state of the enhancer-promoter configuration. Subsequent cell-specific recruitment of transcription factors or epigenetic modifiers would be permissive for gene expression. It is also possible that these interactions are residual or transient in non-expressing cells, therefore interfering with proper gene activation. Interestingly, we have identified at least 5 CNCs spanning a 200 kb genomic region that interact with loci within 10 kb of OLIG2. Moreover, some of these CNCs interact with one another, suggesting that these converge toward the same region of the nucleus. The difference between OLIG2 expressing versus non-expressing cells could be the direct consequence of the type of looping occurring in the region. Interestingly, a recent study suggested that the mouse orthologue of CNC5 has the potential to repress transcription in embryonic stem cells [32].

We have shown that CNCs are able to interact with a number of loci in *trans* on other chromosomes. The most striking observation is that CNCs tend to interact with CNCs. The functional significance of these interactions is unclear although some of these trans-CIRs are enriched for regions near functionally related genes. It is interesting that the various CNCs analyzed in this study display different proportions of *cis* versus *trans* interactions. Cell specificity, the potential functions, as well as the nuclear localization of these DNA elements could affect the amount and type of interactions. Potent enhancer/silencers with strong gene and cell specificity may be expected to make fewer interactions than enhancer lacking spatial/temporal specificity. In addition, chromosomes appear to be preferentially confined in specific regions of the nucleus [33], [34]. Moreover, there is significant intermingling between these chromosome territories [35]. Thus, the position of a locus relative to its chromosome territory may influence the number of potential interactions with neighboring chromosomes. Alternatively, CNCs that regulate genes sharing common transcription regulators could be targeted to the same transcription factory [36], [37]. This could lead to a local increase in concentration of co-regulated loci. Thus, the various numbers of *cis* versus *trans* interactions could reflect the number of loci in such transcription factories. Targeting of regulatory sequences to small number of foci in the nucleus could also explain why we observe enrichment for CNC-CNC interactions.

We have analyzed in this study 10 CNCs. The investigation of a much larger number of these elements may define different classes of CNCs based on criteria that include the type and number of interactions (CNC-CNC or CNC-gene), the cellular and temporal specificity. These classes may in turn correlate with a specific function. For instance, CNCs with strong specificity could be involved directly in gene regulations (e.g. CNC7, 8 and 9) whereas CNCs with less specificity may be important for the formation of chromatin-chromatin interactions (e.g. CNC1, 10).

We have shown that the study of interactions between genomic loci in conjunction with *in vivo* functional assays can provide valuable information not only on the function of CNCs but on the genes they regulate. The elucidation of functional physical interactions among different genomic regions would enhance our understanding of normal development properties and disease states.

## Materials and Methods

### Ethics statement

The human erythroleukemic cell line K562 was obtained from the ATCC repository and the GM06990 lymphoblastoid cell line was obtained from the Coriell cell repository.

### Chromosome conformation capture

Chromosome conformation capture was performed as described [38] with the following modifications. Cells were grown in 50 ml RPMI medium (Invitrogen) supplemented with 10% heat inactivated fetal bovine serum and 100µg/ml streptomycin/penicillin at a concentration of about 2×10^5^ cells/ml (1×10^7^ cells). Cross-linking with formaldehyde (1% v/v) was performed for 10 minutes at room temperature directly in the cell media prior to quenching with 125 mM glycine. Cells were washed twice in ice-cold PBS and lysed for 1 hour on ice with mild stirring in 20 ml 1xTBS-Tween (10 mM Tris-HCl pH 7.5, 3 mM CaCl2, 2 mM MgCl2, 15 mM NaCl, 0.5% v/v Tween 40) supplemented with protease inhibitor (Complete, Roche) and 5 mM PMSF. The cell lysate was homogenized with 15 strokes in a Douncer (‘A’ or tight pestle) and washed with PBS (centrifugation: 1′200 g at 4°C for 10 minutes). The lysate was subsequently resuspended in 5 ml 25% (w/v) sucrose-TBS and underlayed with 5 ml 50% (w/v) sucrose-TBS. Nuclei were pelleted for 20 min (4600 g at 4°C), washed under the same conditions with 5 ml 25% (w/v) sucrose-TBS and resuspended in 500 µl 1.2 x DpnII restriction buffer. Restriction with DpnII, ligation, crosslink reversal and DNA purification were carried out as described previously [39].

The 4C library was generated from 100 ng of ligated DNA with two successive rounds of PCR amplification using 2 nested pairs of primers (Table S2). The PCRs (20pmoles of round A primers) were performed under the following conditions during Round A: 98°C for 30 s, 34 cycles of 98°C for 10 s/65°C for 30 s/72°C for 90 s and followed by a final elongation step at 72°C for 3 min. A 1/100 dilution of the initial PCR product was subsequently amplified with 40pmoles of round B primers (94°C for 3 min, 32 cycles of 94°C for 30 ss/65°C for 30 s/72°C for 90 s and followed by a final elongation step at 72°C for 3 min). The primers used during the second round of amplification have additional nucleotides at their 5′ end (5′-AATGATACGGCGACCACCGA and 5′-CAAGCAGAAGACGGCATACGA). These are required for DNA colony amplification on the cluster station as part of the Illumina Genome Analyzer high-throughput sequencing procedure. The library was gel purified to reduce the amount of DNA originating from self-ligation of the DpnII restricted bait. Sequencing was carried out using an Illumina Genome Analyzer. The sequencing primers were designed to anneal just upstream of the DpnII (GATC) restriction site on one side of the bait. Hence all sequences begin with GATC.

### Sequence analysis

The sequences were aligned against the repeat-masked human genome (build hg18) using blat [40]. The quality of the alignments was filtered according to the following criteria: 1) the minimum match length was set to 29 nucleotides with no gap larger than 1 nucleotide 2) alignments had to start with the DpnII site as the first nucleotides. All sequences that did not fit these criteria or that aligned to more than one location on the genome were discarded.

### Lentivector-mediated transgenesis

Lentiviral vectors were generated by co-transfecting the transfer vector with PMD2G and R8.74 plasmids (http://tronolab.epfl.ch/) in 293T cells. F0 transgenic embryos were generated by perivitelline injection of lentivectors in mouse fertilized oocytes as described in [41].

## Supporting Information

Figure S1 **Chromosomal map showing the position of the baits used in this study**. (See Table S1 for their exact coordinates). CNCs are shown in orange whereas non-CNCs are shown in blue. The image was generated with the UCSC genome browser (http://genome.ucsc.edu/).
(EPS)

Figure S2 **Comparisons of the quantile distributions of the conservations scores of the nearest conservation block.** The “experiment” and “control” distributions correspond to the DpnII fragments in the crosslinked and non-crosslinked 4C libraries respectively, whereas 10000 DpnII fragments were sampled from the genome for the “simulation” distribution. The dashed lines correspond to the expected plot if the distributions are equal. The statistical significance (two sample Wilcoxon test) of the divergence between two distributions is displayed at the bottom-right of each plot.
(EPS)

Figure S3 **Map of interactions of CNC1 to CNC9 in a 1.4 Mb region of human chromosome 21.** The upper track shows the position of all 9 CNCs (red). The nine following tracks correspond to the interactions maps of CNC1 to CNC9. The blue bars correspond to the number of sequences tags (log 2 transformed) found in the library generated with the corresponding CNC. The genes in the region are displayed at the bottom of the map. The image was generated with the UCSC genome browser (http://genome.ucsc.edu/).
(EPS)

Table S1 List showing the human chromosome coordinates (build hg18) of the DpnII fragments used as baits in the 4C experiments described in this study. The log odd score (lod), length (Size) and conservation score (Score) of the most conserved element within the DpnII fragments are also shown (PhastCons conserved element: 17-way vertebrate multiz alignments).
(DOC)

Table S2 List and sequences of primers used for the amplification of the 4C libraries.
(DOC)

Table S3 Overlaps between genes found within 10 kb of all DpnII fragment tags (minimum threshold of 50 sequence tags) in each CNC library and various data sets using the Molecular Signatures Database (MSigDB).
(PDF)

Table S4 **Reproducibility of biological duplicates for CNC1 to CNC10.** The number of DpnII fragments identified for the corresponding CNC in both experiments (1 and 2) is shown. The overlap corresponds to the number of fragments replicated. Reproducibility (Fraction) is expressed as the percentage of the number of overlapping fragments over the number of fragment in experiment 1.
(DOC)

File S1 **List of sequenced DpnII fragments.** The coordinates (Chr, DpnIIStart, DpnIIEnd) of all DpnII fragments associated with the specified library (BaitID) are listed. These coordinates correspond to human genome build hg18. The number of sequencing reads for each fragment is also shown (NumbReads).
(TXT)
