# Supplementary material for: Chromosome Conformation Capture Uncovers Potential Genome-Wide Interactions between Human Conserved Non-Coding Sequences
Source: PLoS One. 2011 Mar 7;6(3):e17634. doi: 10.1371/journal.pone.0017634 (PMC3049788; doi:10.1371/journal.pone.0017634)
Supplement: Table S1 — List showing the human chromosome coordinates (build hg18) of the DpnII fragments used as baits in the 4C experiments described in this study. The log odd score (lod), length (Size) and conservation score (Score) of the most conserved element within the DpnII fragments are also shown (PhastCons conserved element: 17-way vertebrate multiz alignments). (DOC) [file pone.0017634.s004.doc]

**Table S1.**

| **Bait** | **Chromosome** | **Start** | **End** | **lod** | **Size (bp)** | **Score** |
| --- | --- | --- | --- | --- | --- | --- |
| CNC1 | chr21 | 32718140 | 32718560 | 214 | 203 | 525 |
| CNC2 | chr21 | 32836575 | 32837299 | 433 | 305 | 605 |
| CNC3 | chr21 | 33135826 | 33136280 | 827 | 321 | 678 |
| CNC4 | chr21 | 33138765 | 33139332 | 304 | 286 | 565 |
| CNC5 | chr21 | 33212536 | 33213326 | 1375 | 464 | 735 |
| CNC6 | chr21 | 33249354 | 33250686 | 1333 | 530 | 732 |
| CNC7 | chr21 | 33302128 | 33303588 | 1232 | 589 | 723 |
| CNC8 | chr21 | 33389682 | 33391213 | 1388 | 492 | 736 |
| CNC9 | chr21 | 33409685 | 33411690 | 1093 | 485 | 709 |
| CNC10 | chr21 | 39465371 | 39465570 | 1097 | 505 | 710 |
| nonCNC1 | chr21 | 20462676 | 20463552 | n/a | n/a | n/a |
| nonCNC2 | chr21 | 33181340 | 33182055 | n/a | n/a | n/a |
| nonCNC3 | chr21 | 33280627 | 33281536 | n/a | n/a | n/a |
| nonCNC4 | chr21 | 33398043 | 33398342 | n/a | n/a | n/a |
| nonCNC5 | chr21 | 34361121 | 34362336 | n/a | n/a | n/a |
| nonCNC6 | chr21 | 34498667 | 34498995 | n/a | n/a | n/a |
| nonCNC7 | chr21 | 34698528 | 34699475 | n/a | n/a | n/a |
| nonCNC8 | chr21 | 35854084 | 35854620 | n/a | n/a | n/a |
| LCR-HS5 | chr11 | 5267731 | 5269565 | n/a | n/a | n/a |
