# Supplementary material for: Chromosome Conformation Capture Uncovers Potential Genome-Wide Interactions between Human Conserved Non-Coding Sequences
Source: PLoS One. 2011 Mar 7;6(3):e17634. doi: 10.1371/journal.pone.0017634 (PMC3049788; doi:10.1371/journal.pone.0017634)
Supplement: Table S2 — List and sequences of primers used for the amplification of the 4C libraries. (DOC) [file pone.0017634.s005.doc]

**Table S2**

| **Bait/Primer** | **RoundA PCR primers** | **RoundB PCR primers** |
| --- | --- | --- |
| CNC1 / 1 | 5’-TACTGAGGAACAAGAGAAACCCAG | 5’-AATGATACGGCGACCACCGAAACAGGAGATGACAGGTGGAAATG |
| CNC1 / 2 | 5’-CAGATTATTCCCCCACATTGCAGG | 5’-CAAGCAGAAGACGGCATACGAGAATGCTGACAGACTTGAAATGGG |
| CNC2 / 1 | 5’-GGACAAATTTGATGTGTACCAGGG | 5’-AATGATACGGCGACCACCGATCAAGCTGCTGTCACATAATGTGC |
| CNC2 / 2 | 5’-GTGTGTATCCTACACTCTGTGTTC | 5’-CAAGCAGAAGACGGCATACGATCCACCTGGGTGGTGTAACATATC |
| CNC3 / 1 | 5’-CAGCTTAATTGGTTGCAGATTGGG | 5’-AATGATACGGCGACCACCGAATGAATCCTATACCAGGAGCCACC |
| CNC3 / 2 | 5’-CATTGTACCACGGTTATCATGTCC | 5’-CAAGCAGAAGACGGCATACGAAGAACAAGGAGCATGTGATTGAGG |
| CNC4 / 1 | 5’-CCGTGAGGTCTGGAAGATTTAATG | 5’-AATGATACGGCGACCACCGATCATTAAATAACCAGCCTGGCAGC |
| CNC4 / 2 | 5’-AGGAGAGTAGATGGACTCATGATG | 5’-CAAGCAGAAGACGGCATACGACAACTGCCATCTTTTCCTGTTCTC |
| CNC5 / 1 | 5’-AAAACATTACAGCAGGGACAGACG | 5’-AATGATACGGCGACCACCGAACTAATAAGGACCCCTCGCACTAC |
| CNC5 / 2 | 5’-ATGAATTGAGTGTGGTCTGTCTGC | 5’-CAAGCAGAAGACGGCATACGACTTTCTCTGTCACCTAGGCTCTAG |
| CNC6 / 1 | 5’-TACTAAGCCGTGATGTGCTGTTTC | 5’-AATGATACGGCGACCACCGAGCACGTTATCTGACAATGCTACTC |
| CNC6 / 2 | 5’-AAGAACTTGCTGGTAGGTTTCAGC | 5’-CAAGCAGAAGACGGCATACGAGTATTTTGGCAGGAGAAATGCTCC |
| CNC7 / 1 | 5’-CTCGCATCTTCCACTTGGTATGAG | 5’-AATGATACGGCGACCACCGATAAGGAGACAGCATAGGCATGTTC |
| CNC7 / 2 | 5’-GGAAGCAATATCATAGCCATGTGC | 5’-CAAGCAGAAGACGGCATACGAGCAAACAGGTCTAAGCATTGGGAC |
| CNC8 / 1 | 5’-TTCGGGTCCATAAAAGTCCCTTGC | 5’-AATGATACGGCGACCACCGAAATGAGCCCAGCCTTTGAACCTAC |
| CNC8 / 2 | 5’-TGGGGTCTTCTTATTGCTGTCTTG | 5’-CAAGCAGAAGACGGCATACGAAGGACCAATTGCATTATTGCCACG |
| CNC9 / 1 | 5’-GGGACACCAGCACAATTGAATCTG | 5’-AATGATACGGCGACCACCGAAAGCCCAAGTGAGATAGGACTATG |
| CNC9 / 2 | 5’-CTCTCACTTCCCACAACTTCTCTC | 5’-CAAGCAGAAGACGGCATACGAAAGGCAGGTGACCTAAGTATCGTG |
| CNC10 / 1 | 5’-TCCAACACACTCAATGATGGACTG | 5’-AATGATACGGCGACCACCGATGAGCCCTGTACTCTCTCGTATTG |
| CNC10 / 2 | 5’-TCTGTGTGGTGTTGGAGTCTTC | 5’-CAAGCAGAAGACGGCATACGAATACTGGTCCAAGCTGTCCAATTC |
| non-CNC1 / 1 | 5’-TCTATGTGGCTATTTCCAGTTC | 5’-AATGATACGGCGACCACCGAGTTTTTCTAGCTACTCTTCTCC |
| non-CNC1 / 2 | 5’-CTTGGATTTTCCATGCAGCGTTAC | 5’-CAAGCAGAAGACGGCATACGACTTGGATTTTCCATGCAGCGTTAC |
| non-CNC2 / 1 | 5’-AAGGGAGACAGTATTGCTTGGTCC | 5’-AATGATACGGCGACCACCGATCCCACTTATCTGTCTGGGAACTG |
| non-CNC2 / 2 | 5’-CTGTTTCAGTCTGTCTGTTAGTTC | 5’-CAAGCAGAAGACGGCATACGATGGAAAAGTGTGGGGTTTCACAGC |
| non-CNC3 / 1 | 5’-TCAGGGCAACATGCAAGAGAGAAG | 5’-AATGATACGGCGACCACCGAAATGTAAAGAAAGGGGCCTGGCAG |
| non-CNC3 / 2 | 5’-TGTAGAGGTGGTGCAGAAAAATTC | 5’-CAAGCAGAAGACGGCATACGATAGTGGTGGTGAGGTAGTTCGTTC |
| non-CNC4 / 1 | 5’-TTGGGAATTTAGAGCAGAAATCGC | 5’-AATGATACGGCGACCACCGAAAGCAATGTGGGAGCAGTTTTGAC |
| non-CNC4 / 2 | 5’-TAGTACATTGCCAGGGTTTCCAAG | 5’-CAAGCAGAAGACGGCATACGATGTTGCTTTGTCTTAAGAGCAAGG |
| non-CNC5 / 1 | 5’-AGAATCAAAGCAAATGGGCATGGC | 5’-AATGATACGGCGACCACCGAAAGAAGGTCTGAAAGGTGGGTGAG |
| non-CNC5 / 2 | 5’-TTGTTCTGGCCACATGCACCTTTG | 5’-CAAGCAGAAGACGGCATACGACCACAGCTCCTAATGTAAGGCTTC |
| non-CNC6 / 1 | 5’-AAAAAATGGAATCCAGTGTCCAGG | 5’-AATGATACGGCGACCACCGACTCAGTCTCATGTCTTTAATAGGC |
| non-CNC6 / 2 | 5’-TATGCAACTCAGAATCACTCAGTC | 5’-CAAGCAGAAGACGGCATACGATCGCAAACAAGTAAAGCACCTACC |
| non-CNC7 / 1 | 5’-TAACTTGAACTCTCAGGACACGGG | 5’-AATGATACGGCGACCACCGAACTCAATACGTGAGTGTTGCTGCC |
| non-CNC7 / 2 | 5’-CAAACTACTTCTGGCTCCTTGTGC | 5’-CAAGCAGAAGACGGCATACGAAAGTGAACCCCAGTCAAAAGCTCC |
| non-CNC8 / 1 | 5’-TAAGAAAGACTAGGCGTAGTCCCG | 5’-AATGATACGGCGACCACCGAATGTCTCCAACCCAGGTTTGCAAC |
| non-CNC8 / 2 | 5’-AGCTAGGTTCAAATGGGTTACCCG | 5’-CAAGCAGAAGACGGCATACGAGTGCATTAGCTGGAAGTTACTAAC |
| LCR-HS5 / 1 | 5’-CATAGCTGTCTCCCTACCTTGTTC | 5’-AATGATACGGCGACCACCGATCCTGTCCAGACCAAAGTACCTTG |
| LCR-HS5 / 2 | 5’-CTCAGGTTATTCTGTGACCAACAG | 5’-CAAGCAGAAGACGGCATACGAGTGGGAAAAATCAGAGAAGGAGGC |
