# Supplementary material for: Chromosome Conformation Capture Uncovers Potential Genome-Wide Interactions between Human Conserved Non-Coding Sequences
Source: PLoS One. 2011 Mar 7;6(3):e17634. doi: 10.1371/journal.pone.0017634 (PMC3049788; doi:10.1371/journal.pone.0017634)
Supplement: Table S3 — Overlaps between genes found within 10 kb of all DpnII fragment tags (minimum threshold of 50 sequence tags) in each CNC library and various data sets using the Molecular Signatures Database (MSigDB). (PDF) [file pone.0017634.s006.pdf]

CNC1

# genesets in collections:	1454
# genes in comparison (n):	44
# genes in collections (N):	8299

geneset name	# genes in geneset (K)	# genes in overlap (k)	k/K	p value
RHO_GUANYL_NUCLEOTIDE_EXCHANGE_FACTOR_ACTIVITY	12	2	0.1667	**1.72E-03**
GLUTAMATE_SIGNALING_PATHWAY	17	2	0.1176	**3.46E-03**
RAS_GUANYL_NUCLEOTIDE_EXCHANGE_FACTOR_ACTIVITY	19	2	0.1053	**4.31E-03**
GLUTAMATE_RECEPTOR_ACTIVITY	20	2	0.1	**4.76E-03**
DNA_PACKAGING	35	2	0.0571	**1.38E-02**
SH3_SH2_ADAPTOR_ACTIVITY	43	2	0.0465	**2.01E-02**
GUANYL_NUCLEOTIDE_EXCHANGE_FACTOR_ACTIVITY	47	2	0.0426	**2.36E-02**
CYTOPLASM	2137	6	0.0028	**2.49E-02**
MOLECULAR_ADAPTOR_ACTIVITY	49	2	0.0408	**2.54E-02**
CYTOPLASMIC_PART	1385	3	0.0022	**3.43E-02**

Gene/geneset overlap matrix

overlap matrix by gene and geneset	RHO_GUANYL_NUCLEOTIDE_EXCHANGE_FACTOR_ACTIVITY	GLUTAMATE_SIGNALING_PATHWAY	RAS_GUANYL_NUCLEOTIDE_EXCHANGE_FACTOR_ACTIVITY	GLUTAMATE_RECEPTOR_ACTIVITY	DNA_PACKAGING	SH3_SH2_ADAPTOR_ACTIVITY	GUANYL_NUCLEOTIDE_EXCHANGE_FACTOR_ACTIVITY	CYTOPLASM	MOLECULAR_ADAPTOR_ACTIVITY	CYTOPLASMIC_PART	Entrez	Source	description
DOCK2	■		■				■					S	dedicator of cytokinesis 2
TIAM1	■		■				■					S	T-cell lymphoma invasion and metastasis 1
GRIK1		■		■								S	glutamate receptor, ionotropic, kainate 1
GRM5		■		■								S	glutamate receptor, metabotropic 5
DFFB					■			■		■		S	DNA fragmentation factor, 40kDa, beta polypeptide (caspase-activated DNase)
HELLS					■							S	helicase, lymphoid-specific
SH3BGR						■		■	■			S	SH3 domain binding glutamic acid-rich protein
SHB						■			■			S	Src homology 2 domain containing adaptor protein B
SLC9A6								■		■		S	solute carrier family 9 (sodium/hydrogen exchanger), member 6
DC2								■		■		S	-
ATXN1								■				S	ataxin 1
ASNA1								■				S	arsA arsenite transporter, ATP-binding, homolog 1 (bacterial)
PRKG1												S	protein kinase, cGMP-dependent, type I
UBE2L3												S	ubiquitin-conjugating enzyme E2L 3
DSCAM												S	Down syndrome cell adhesion molecule
EFNB2												S	ephrin-B2
TSC22D3												S	TSC22 domain family, member 3
NDFIP1												S	Nedd4 family interacting protein 1
FGF10												S	fibroblast growth factor 10
NFIB												S	nuclear factor I/B
ADK												S	adenosine kinase
DFNA5												S	deafness, autosomal dominant 5
ATP8A2												S	ATPase, aminophospholipid transporter-like, Class I, type 8A, member 2
ODF2												S	outer dense fiber of sperm tails 2
AHCTF1												S	AT hook containing transcription factor 1
ARHGAP24												S	Rho GTPase activating protein 24
BCAS3												S	breast carcinoma amplified sequence 3
C10ORF107												S	chromosome 10 open reading frame 107
C1ORF43												S	chromosome 1 open reading frame 43
C21ORF63												S	chromosome 21 open reading frame 63
CAMTA1												S	calmodulin binding transcription activator 1
CDGAP												S	-
CDH22												S	cadherin-like 22
CHD9												S	chromodomain helicase DNA binding protein 9
CRTC3												S	CREB regulated transcription coactivator 3
E2F5												S	E2F transcription factor 5, p130-binding
G6PC2												S	glucose-6-phosphatase, catalytic, 2
GPR158												S	G protein-coupled receptor 158
HYDIN												S	hydrocephalus inducing homolog (mouse)
MESP2												S	mesoderm posterior 2 homolog (mouse)
SHOC2												S	soc-2 suppressor of clear homolog (C. elegans)
TCP10L												S	t-complex 10 (mouse)-like
UBAP2L												S	ubiquitin associated protein 2-like
ZNRF3												S	zinc and ring finger 3

CNC2

# genesets in collections:	1454
# genes in comparison (n):	13
# genes in collections (N):	8299

geneset name	# genes in geneset (K)	# genes in overlap (k)	k/K	p value
INTEGRAL_TO_MEMBRANE	1332	4	0.003	9.82E-02
INTRINSIC_TO_MEMBRANE	1350	4	0.003	1.01E-01
MULTICELLULAR_ORGANISMAL_DEVELOPMENT	1051	3	0.0029	1.50E-01
MEMBRANE_PART	1673	4	0.0024	1.56E-01
MEMBRANE	1998	4	0.002	2.02E-01
PLASMA_MEMBRANE	1429	3	0.0021	2.21E-01
SYSTEM_DEVELOPMENT	863	2	0.0023	2.52E-01
SIGNAL_TRANSDUCTION	1637	2	0.0012	2.71E-01
INTEGRAL_TO_PLASMA_MEMBRANE	978	2	0.002	2.73E-01
INTRINSIC_TO_PLASMA_MEMBRANE	992	2	0.002	2.75E-01

Gene/geneset overlap matrix

overlap matrix by gene and geneset	INTEGRAL_TO_MEMBRANE	INTRINSIC_TO_MEMBRANE	MULTICELLULAR_ORGANISMAL_DEVELOPMENT	MEMBRANE_PART	MEMBRANE	PLASMA_MEMBRANE	SYSTEM_DEVELOPMENT	SIGNAL_TRANSDUCTION	INTEGRAL_TO_PLASMA_MEMBRANE	INTRINSIC_TO_PLASMA_MEMBRANE	Entrez	Source	description
GRIK1	■	■	■	■	■	■	■	■	■	■		S	glutamate receptor, ionotropic, kainate 1
MMP16	■	■		■	■	■			■	■		S	matrix metallopeptidase 16 (membrane-inserted)
NCAM2	■	■		■	■	■						S	neural cell adhesion molecule 2
SYNE1	■	■		■	■							S	spectrin repeat containing, nuclear envelope 1
LAMA3			■				■					S	laminin, alpha 3
HUNK			■					■				S	hormonally upregulated Neu-associated kinase
SEPT7												S	septin 7
SYNJ1												S	synaptojanin 1
GAS7												S	growth arrest-specific 7
C21ORF63												S	chromosome 21 open reading frame 63
LRRC8B												S	leucine rich repeat containing 8 family, member B
TCP10L												S	t-complex 10 (mouse)-like
TP53TG3												S	-

CNC3

genesets in collections: 1454
genes in comparison (n): 12
genes in collections (N): 8299

geneset name	# genes in geneset (K)	# genes in overlap (k)	k/K	p value
DNA_DAMAGE_RESPONSE__SIGNAL_TRANSDUCTION	35	2	0.0571	**1.10E-03**
HYDROLASE_ACTIVITY__HYDROLYZING_O_GLYCOSYL_COMPOU	37	2	0.0541	**1.22E-03**
HYDROLASE_ACTIVITY__ACTING_ON_GLYCOSYL_BONDS	47	2	0.0426	**1.96E-03**
MAGNESIUM_ION_BINDING	63	2	0.0317	**3.48E-03**
MITOTIC_CELL_CYCLE	153	2	0.0131	**1.85E-02**
RESPONSE_TO_DNA_DAMAGE_STIMULUS	162	2	0.0123	**2.06E-02**
CELL_CYCLE_PHASE	170	2	0.0118	**2.24E-02**
CELL_CYCLE_PROCESS	193	2	0.0104	**2.81E-02**
RESPONSE_TO_ENDOGENOUS_STIMULUS	200	2	0.01	**3.00E-02**
PROTEIN_MODIFICATION_PROCESS	632	3	0.0047	**4.76E-02**

Gene/geneset overlap matrix

overlap matrix by gene and geneset	DNA_DAMAGE_RESPONSE__SIGNAL_TRANSDUCTION	HYDROLASE_ACTIVITY__HYDROLYZING_O_GLYCOSYL_COMPOUNDS	HYDROLASE_ACTIVITY__ACTING_ON_GLYCOSYL_BONDS	MAGNESIUM_ION_BINDING	MITOTIC_CELL_CYCLE	RESPONSE_TO_DNA_DAMAGE_STIMULUS	CELL_CYCLE_PHASE	CELL_CYCLE_PROCESS	RESPONSE_TO_ENDOGENOUS_STIMULUS	PROTEIN_MODIFICATION_PROCESS	Entrez	Source	description
ABL1	■			■	■	■	■	■	■	■		S	v-abl Abelson murine leukemia viral oncogene homolog 1
BRSK1	■			■	■	■	■	■	■	■		S	BR serine/threonine kinase 1
MAN2B1		■	■							■		S	mannosidase, alpha, class 2B, member 1
HPSE2		■	■									S	heparanase 2
IL15												S	interleukin 15
GRIN2A												S	glutamate receptor, ionotropic, N-methyl D-aspartate 2A
NFIB												S	nuclear factor I/B
SYNJ1												S	synaptojanin 1
C20ORF74												S	chromosome 20 open reading frame 74
MRPS6												S	mitochondrial ribosomal protein S6
OLIG1												S	oligodendrocyte transcription factor 1
RNF122												S	ring finger protein 122

CNC4

genesets in collections: 1454
genes in comparison (n): 14
genes in collections (N): 8299

geneset name	# genes in geneset (K)	# genes in overlap (k)	k/K	p value
CYTOPLASM	2137	3	0.0014	2.35E-01

Gene/geneset overlap matrix

overlap matrix by gene and geneset	CYTOPLASM	Entrez	Source	description
SEPT2	■		S	septin 2
FH	■		S	fumarate hydratase
HMGCS1	■		S	3-hydroxy-3-methylglutaryl-Coenzyme A synthase 1 (soluble)
HDLBP			S	high density lipoprotein binding protein (vigilin)
POU2AF1			S	POU domain, class 2, associating factor 1
MAPK10			S	mitogen-activated protein kinase 10
SYNJ1			S	synaptojanin 1
PHKB			S	phosphorylase kinase, beta
C21ORF62			S	chromosome 21 open reading frame 62
C21ORF66			S	chromosome 21 open reading frame 66
C7ORF42			S	chromosome 7 open reading frame 42
DIAPH3			S	diaphanous homolog 3 (Drosophila)
HEPHL1			S	hephaestin-like 1
OLIG1			S	oligodendrocyte transcription factor 1

CNC5

# genesets in collections:	1454
# genes in comparison (n):	15
# genes in collections (N):	8299

geneset name	# genes in geneset (K)	# genes in overlap (k)	k/K	p value
REPLICATION_FORK	18	2	0.1111	**4.55E-04**
DOUBLE_STRAND_BREAK_REPAIR	23	2	0.087	**7.46E-04**
DNA_REPAIR	125	3	0.024	**1.27E-03**
RESPONSE_TO_DNA_DAMAGE_STIMULUS	162	3	0.0185	**2.63E-03**
DNA_DEPENDENT_DNA_REPLICATION	56	2	0.0357	**4.31E-03**
RESPONSE_TO_ENDOGENOUS_STIMULUS	200	3	0.015	**4.70E-03**
HEMOPOIESIS	75	2	0.0267	**7.54E-03**
HEMOPOIETIC_OR_LYMPHOID_ORGAN_DEVELOPMENT	77	2	0.026	**7.93E-03**
IMMUNE_SYSTEM_DEVELOPMENT	81	2	0.0247	**8.72E-03**
DNA_METABOLIC_PROCESS	257	3	0.0117	**9.20E-03**

Gene/geneset overlap matrix

overlap matrix by gene and geneset	REPLICATION_FORK	DOUBLE_STRAND_BREAK_REPAIR	DNA_REPAIR	RESPONSE_TO_DNA_DAMAGE_STIMULUS	DNA_DEPENDENT_DNA_REPLICATION	RESPONSE_TO_ENDOGENOUS_STIMULUS	HEMOPOIESIS	HEMOPOIETIC_OR_LYMPHOID_ORGAN_DEVELOPMENT	IMMUNE_SYSTEM_DEVELOPMENT	DNA_METABOLIC_PROCESS	Entrez	Source	description
POLA1	■	■	■	■	■	■				■		S	polymerase (DNA directed), alpha 1
RFC3	■		■	■	■	■				■		S	replication factor C (activator 1) 3, 38kDa
NHEJ1		■	■	■		■	■	■	■	■		S	nonhomologous end-joining factor 1
ACVR2A							■	■	■			S	activin A receptor, type IIA
CLIC5												S	chloride intracellular channel 5
ACYP2												S	acylphosphatase 2, muscle type
HPCAL4												S	hippocalcin like 4
SYNJ1												S	synaptojanin 1
C21ORF62												S	chromosome 21 open reading frame 62
KSR1												S	kinase suppressor of ras 1
MGAT4C												S	mannosyl (alpha-1,3-)-glycoprotein beta-1,4-N-acetylglucosaminyltransferase, isozyme C (putative)
MRAP												S	melanocortin 2 receptor accessory protein
NAP5												S	-
OLIG2												S	oligodendrocyte lineage transcription factor 2
PXDN												S	peroxidasin homolog (Drosophila)

CNC6

# genesets in collections:	1454
# genes in comparison (n):	10
# genes in collections (N):	8299

geneset name	# genes in geneset (K)	# genes in overlap (k)	k/K	p value
VESICLE_MEDIATED_TRANSPORT	194	3	0.0155	**1.28E-03**
MEMBRANE_ORGANIZATION_AND_BIOGENESIS	135	2	0.0148	**1.04E-02**
GOLGI_APPARATUS	227	2	0.0088	**2.69E-02**
TRANSPORT	796	3	0.0038	5.22E-02
ESTABLISHMENT_OF_LOCALIZATION	872	3	0.0034	6.39E-02
MEMBRANE	1998	4	0.002	1.35E-01
CYTOPLASM	2137	4	0.0019	1.55E-01
PROTEIN_METABOLIC_PROCESS	1232	2	0.0016	2.74E-01
CYTOPLASMIC_PART	1385	2	0.0014	2.91E-01
NUCLEUS	1433	2	0.0014	2.95E-01

Gene/geneset overlap matrix

overlap matrix by gene and geneset	VESICLE_MEDIATED_TRANSPORT	MEMBRANE_ORGANIZATION_AND_BIOGENESIS	GOLGI_APPARATUS	TRANSPORT	ESTABLISHMENT_OF_LOCALIZATION	MEMBRANE	CYTOPLASM	PROTEIN_METABOLIC_PROCESS	CYTOPLASMIC_PART	NUCLEUS	Entrez	Source	description
SYNJ1	■	■		■	■							S	synaptojanin 1
ITSN1	■	■		■	■							S	intersectin 1 (SH3 domain protein)
DOPEY2	■		■	■	■	■	■		■			S	dopey family member 2
FUT9			■				■	■	■			S	fucosyltransferase 9 (alpha (1,3) fucosyltransferase)
FRAP1						■	■	■				S	FK506 binding protein 12-rapamycin associated protein 1
UBIAD1						■	■			■		S	UbiA prenyltransferase domain containing 1
NCAM2						■						S	neural cell adhesion molecule 2
HIC2										■		S	hypermethylated in cancer 2
GNG7												S	guanine nucleotide binding protein (G protein), gamma 7
OLIG2												S	oligodendrocyte lineage transcription factor 2

CNC7

Only 2 genes in this set
OLIG2, RCAN1

CNC8

Only 3 genes in this set
IFNGR2, OLIG2, TNRC6B

CNC9

genesets in collections: 1454
genes in comparison (n): 6
genes in collections (N): 8299

geneset name	# genes in geneset (K)	# genes in overlap (k)	k/K	p value
MEMBRANE_ORGANIZATION_AND_BIOGENESIS	135	2	0.0148	**3.69E-03**
VESICLE_MEDIATED_TRANSPORT	194	2	0.0103	**7.43E-03**
TRANSPORT	796	2	0.0025	9.22E-02
ESTABLISHMENT_OF_LOCALIZATION	872	2	0.0023	1.06E-01
PLASMA_MEMBRANE	1429	2	0.0014	2.09E-01
MEMBRANE	1998	2	0.001	2.89E-01

Gene/geneset overlap matrix

overlap matrix by gene and geneset	MEMBRANE_ORGANIZATION_AND_BIOGENESIS	VESICLE_MEDIATED_TRANSPORT	TRANSPORT	ESTABLISHMENT_OF_LOCALIZATION	PLASMA_MEMBRANE	MEMBRANE	Entrez	Source	description
SYNJ1	■	■	■	■				S	synaptojanin 1
ITSN1	■	■	■	■				S	intersectin 1 (SH3 domain protein)
TMEM50B					■	■		S	transmembrane protein 50B
IFNAR1					■	■		S	interferon (alpha, beta and omega) receptor 1
ZRANB2								S	zinc finger, RAN-binding domain containing 2
OLIG2								S	oligodendrocyte lineage transcription factor 2

CNC10

genesets in collections: 1454
genes in comparison (n): 74
genes in collections (N): 8299

geneset name	# genes in geneset (K)	# genes in overlap (k)	k/K	p value
NUCLEOBASE NUCLEOSIDE NUCLEOTIDE AND NUCLEIC A(	1246	2	0.0016	**4.80E-04**
INTRINSIC_TO_MEMBRANE	1350	3	0.0022	**9.04E-04**
INTEGRAL_TO_MEMBRANE	1332	3	0.0023	**1.04E-03**
INTRINSIC_TO_PLASMA_MEMBRANE	992	2	0.002	**3.93E-03**
INTEGRAL_TO_PLASMA_MEMBRANE	978	2	0.002	**4.39E-03**
NUCLEUS	1433	5	0.0035	**5.04E-03**
RNA_METABOLIC_PROCESS	843	2	0.0024	**1.22E-02**
BIOPOLYMER_METABOLIC_PROCESS	1687	8	0.0047	**1.33E-02**
MEMBRANE_PART	1673	8	0.0048	**1.43E-02**
CYTOPLASM	2137	12	0.0056	**1.77E-02**

Gene/geneset overlap matrix

overlap matrix by gene and geneset	NUCLEOBASE__NUCLEOSIDE__NUCLEOTIDE_AND_NUCLEIC_ACID_METABOLIC_PROCESS	INTRINSIC_TO_MEMBRANE	INTEGRAL_TO_MEMBRANE	INTRINSIC_TO_PLASMA_MEMBRANE	INTEGRAL_TO_PLASMA_MEMBRANE	NUCLEUS	RNA_METABOLIC_PROCESS	BIOPOLYMER_METABOLIC_PROCESS	MEMBRANE_PART	CYTOPLASM	Entrez	Source	description
NR6A1	■					■	■	■				S	nuclear receptor subfamily 6, group A, member 1
PEX14	■						■	■		■		S	peroxisomal biogenesis factor 14
TSPAN9		■	■	■	■				■			S	tetraspanin 9
MMP16		■	■	■	■				■			S	matrix metallopeptidase 16 (membrane-inserted)
VAT1		■	■						■			S	vesicle amine transport protein 1 homolog (T californica)
ERG						■		■				S	v-ets erythroblastosis virus E26 oncogene homolog (avian)
PDZD2						■			■	■		S	PDZ domain containing 2
BUB1B						■						S	BUB1 budding uninhibited by benzimidazoles 1 homolog beta (yeast)
BRD1						■						S	bromodomain containing 1
MAP3K12								■		■		S	mitogen-activated protein kinase kinase kinase 12
MAP4K4								■				S	mitogen-activated protein kinase kinase kinase kinase 4
PRKCE								■				S	protein kinase C, epsilon
PRKG1								■				S	protein kinase, cGMP-dependent, type I
CAMK4								■				S	calcium/calmodulin-dependent protein kinase IV
SYTL2									■	■		S	synaptotagmin-like 2
ATP5O									■	■		S	ATP synthase, H+ transporting, mitochondrial F1 complex, O subunit (oligomycin sensitivity conferring protein)
COPB2									■	■		S	coatomer protein complex, subunit beta 2 (beta prime)
LIMA1									■			S	LIM domain and actin binding 1
STRN4										■		S	striatin, calmodulin binding protein 4
NEK2										■		S	NIMA (never in mitosis gene a)-related kinase 2
TRAK2										■		S	trafficking protein, kinesin binding 2
MRPS22										■		S	mitochondrial ribosomal protein S22
ME3										■		S	malic enzyme 3, NADP(+)-dependent, mitochondrial
USH2A										■		S	Usher syndrome 2A (autosomal recessive, mild)
CALD1												S	caldesmon 1
RYR2												S	ryanodine receptor 2 (cardiac)
KCNJ12												S	potassium inwardly-rectifying channel, subfamily J, member 12
CSNK2A2												S	casein kinase 2, alpha prime polypeptide
CDKL2												S	cyclin-dependent kinase-like 2 (CDC2-related kinase)
MAPRE2												S	microtubule-associated protein, RP/EB family, member 2
RND2												S	Rho family GTPase 2
CYP2C9												S	cytochrome P450, family 2, subfamily C, polypeptide 9
AK2												S	adenylate kinase 2
CLTCL1												S	clathrin, heavy chain-like 1

AK2												S	adenylate kinase 2
CLTCL1												S	clathrin, heavy chain-like 1
ITSN1												S	intersectin 1 (SH3 domain protein)
CXCL13												S	chemokine (C-X-C motif) ligand 13 (B-cell chemoattractant)
CD9												S	CD9 molecule
DLX6												S	distal-less homeobox 6
COL19A1												S	collagen, type XIX, alpha 1
SEMA5A												S	sema domain, seven thrombospondin repeats (type 1 and type 1-like), transmembrane domain (TM) and short cytoplasmic domain, (semaphorin) 5A
MEOX2												S	mesenchyme homeobox 2
SCML2												S	sex comb on midleg-like 2 (Drosophila)
RFX1												S	regulatory factor X, 1 (influences HLA class II expression)
CFI												S	complement factor I
FNBP1												S	formin binding protein 1
SNX9												S	sorting nexin 9
PCBP2												S	poly(rC) binding protein 2
MANBA												S	mannosidase, beta A, lysosomal
ASTN2												S	astrotactin 2
AUTS2												S	autism susceptibility candidate 2
BMP2K												S	BMP2 inducible kinase
C3ORF21												S	chromosome 3 open reading frame 21
C6ORF142												S	chromosome 6 open reading frame 142
C9ORF24												S	chromosome 9 open reading frame 24
C9ORF66												S	chromosome 9 open reading frame 66
CA10												S	carbonic anhydrase X
CACNG3												S	calcium channel, voltage-dependent, gamma subunit 3
CAPSL												S	calcyphosine-like
CPNE5												S	copine V
FMN2												S	formin 2
FRMD4A												S	FERM domain containing 4A
G6PC2												S	glucose-6-phosphatase, catalytic, 2
KIAA1161												S	KIAA1161
KIAA1211												S	-
LMBRD2												S	LMBR1 domain containing 2
PRKD2												S	protein kinase D2
PSMA8												S	proteasome (prosome, macropain) subunit, alpha type, 8
RPIA												S	ribose 5-phosphate isomerase A (ribose 5-phosphate epimerase)
SFT2D2												S	SFT2 domain containing 2
ST7												S	suppression of tumorigenicity 7
TASP1												S	taspase, threonine aspartase, 1
TRIM4												S	tripartite motif-containing 4
TSPAN19												S	tetraspanin 19
ZNRF1												S	zinc and ring finger 1
