# Supplementary material for: Chromosome Conformation Capture Uncovers Potential Genome-Wide Interactions between Human Conserved Non-Coding Sequences
Source: PLoS One. 2011 Mar 7;6(3):e17634. doi: 10.1371/journal.pone.0017634 (PMC3049788; doi:10.1371/journal.pone.0017634)
Supplement: Table S4 — Reproducibility of biological duplicates for CNC1 to CNC10. The number of DpnII fragments identified for the corresponding CNC in both experiments (1 and 2) is shown. The overlap corresponds to the number of fragments replicated. Reproducibility (Fraction) is expressed as the percentage of the number of overlapping fragments over the number of fragment in experiment 1. (DOC) [file pone.0017634.s007.doc]

**Table S4.**

| **Bait** | **Experiment 1** | **Experiment 2** | **Overlap** | **Fraction (%)** |
| --- | --- | --- | --- | --- |
| CNC1 | 117 | 149 | 103 | 88.0 |
| CNC2 | 1123 | 1263 | 603 | 53.7 |
| CNC3 | 22 | 47 | 10 | 45.5 |
| CNC4 | 25 | 65 | 21 | 84.0 |
| CNC5 | 23 | 45 | 18 | 78.3 |
| CNC6 | 45 | 48 | 35 | 77.8 |
| CNC7 | 9 | 10 | 6 | 66.7 |
| CNC8 | 16 | 23 | 7 | 43.8 |
| CNC9 | 15 | 24 | 11 | 73.3 |
| CNC10 | 98 | 191 | 9 | 9.2 |
